# Anticancer activity and antibody-dependent cell-mediated cytotoxicity of novel anti-nucleolin antibodies

**DOI:** 10.1038/s41598-018-25816-8

**Published:** 2018-05-10

**Authors:** Sofia Romano, Vera Moura, Sérgio Simões, João Nuno Moreira, João Gonçalves

**Affiliations:** 1 CNC – Center for Neuroscience and Cell Biology, Faculty of Medicine (Pólo I), University of Coimbra, Rua Larga, 3004-504 Coimbra, Portugal; 2 IIIUC – Institute for Interdisciplinary Research, University of Coimbra, Casa Costa Alemão – Pólo II, Rua Dom Francisco de Lemos, 3030-789 Coimbra, Portugal; 3 TREAT U, SA, Parque Industrial de Taveiro, Lote 44, 3045-508 Coimbra, Portugal; 4 FFUC – Faculty of Pharmacy, University of Coimbra, Pólo das Ciências da Saúde, Azinhaga de Santa Comba, 3000-548 Coimbra, Portugal; 5 iMed.ULisboa - Research Institute for Medicines, Faculty of Pharmacy, University of Lisbon, Avenida Prof. Gama Pinto, 1649-003 Lisbon, Portugal

## Abstract

Nucleolin arises as a relevant target for cancer therapy, as it is overexpressed at the surface of cancer and angiogenic endothelial cells thus enabling a dual cellular targeting strategy. Immunotherapeutic strategies, albeit of proven therapeutic relevance, have been scarcely explored against this target. Therefore, this work aimed at engineering an anti-nucleolin VHH-based antibody capable of triggering anticancer immune responses. Herein, anti-nucleolin VHHs have been generated upon grafting F3 peptide-derived nucleolin-binding sequences onto a VHH CDR1 or CDR3. One of these nucleolin-binding CDR3-grafted VHH was subsequently fused to a human IgG1 Fc region, enabling a significant antibody-dependent cell-mediated cytotoxicity (ADCC). The generated anti-nucleolin VHH revealed increased binding and antiproliferative effects against cancer cells, relative to the parental VHH, while the VHH-Fc counterpart presented increased cytotoxicity relative to the corresponding VHH. This VHH-Fc also triggered an ADCC effect, in the nanomolar range, against a nucleolin-overexpressing cancer cell line. This effect was evidenced by a 2 or 1.7-fold increase of cell death, in the presence of PBMCs, relative to the parental VHH-Fc or the VHH counterpart, respectively. Overall, these formats represent the first anti-nucleolin VHHs and the first anti-nucleolin antibody with ADCC activity that have been successfully developed.

## Introduction

Nucleolin is a multifunctional protein expressed in the nucleus of exponentially growing eukaryotic cells, where it participates in rRNA synthesis and ribosome biogenesis^1^. However, in highly proliferating cells, such as cancer cells and angiogenic endothelial cells of the tumour vasculature, nucleolin is translocated to the surface^2^. This translocation makes nucleolin a potential target for anticancer therapy, as it is accessible to drugs administered intravenously, namely the one overexpressed in the tumour vasculature^3^. In addition, as nucleolin interacts with proteins involved in cell proliferation and migration pathways (such as EGFR^4^ and CXCR4^5^). As such, nucleolin-based targeting strategies might also disrupt the referred pathways, thus compromising tumour progression^6–11^.

Antibodies are nowadays one of the major classes of therapeutics and are currently used against several malignancies. These proteins combine a high affinity to their targets through the variable domains (VH and VL) of the antigen binding fragment (Fab), with the capacity to trigger cell death by several mechanisms. These include direct cell death (upon interfering with the signalling pathways in which the target is involved) and immune responses, mediated by the Fc region. One of these immune responses is antibody-dependent cell-mediated cytotoxicity (ADCC)^12^, which plays a relevant role in the therapeutic outcome of antibodies currently used in the clinic, such as cetuximab, trastuzumab and rituximab^13–18^.

Although antibodies have been a breakthrough in cancer therapy, some of their properties constitute a drawback, as the high molecular weight (around 150 kDa). In this respect, the tumor penetration of smaller antibody variants is expected to take place in a higher extent, while maintaining long circulating time in the blood. The relevance of these features on the overall pharmacodynamics, has led to the development of smaller antibody formats^19^. In camelids, non-canonical antibodies (HCabs) have been identified, whose antigen binding fragment is solely composed by the heavy chain variable domain, named VHH. This results in antibodies of approximately 80 kDa^20^, a molecular size that has enabled higher tumour/blood accumulation ratio, relative to a full-length IgG (150 kD), a scFv (28 kDa) and a diabody (55 kDa), and increased tumour accumulation relative to full-length IgG and a Fab’2 fragment (fusion of two Fab fragments, 110 kDa)^19^.

Nucleolin targeting has been widely explored for the delivery of cytotoxic drugs by nanoparticles, using either the nucleolin-binding F3 peptide or the aptamer AS1411^21^. In addition, different nucleolin ligands have shown antiproliferative and/or anti-angiogenic properties, both *in vitro* and *in vivo*, including AS144^11^, pseudopeptides^6,8,10,21^ and a rabbit antibody^22^. Recently, an anti-nucleolin scFv (fused VH and VL domains, ≈35 kDa) has also been generated^23^ and further fused to a RNase, thus becoming cytotoxic upon internalization^24^. However, the potential of nucleolin as a target to enable antitumour immune responses remains unexplored. Herein it is hypothesized that generation of anti-nucleolin antibodies, generated upon grafting of the nucleolin binding domain from the F3 peptide, will enable increased cytotoxicity against nucleolin-overexpressing cells and, upon further fusion to a human IgG1 Fc region, will induce an ADCC effect.

## Results

### Binding of VHHs grafted with a F3 peptide-derived sequence to human nucleolin

**Figure 1:**
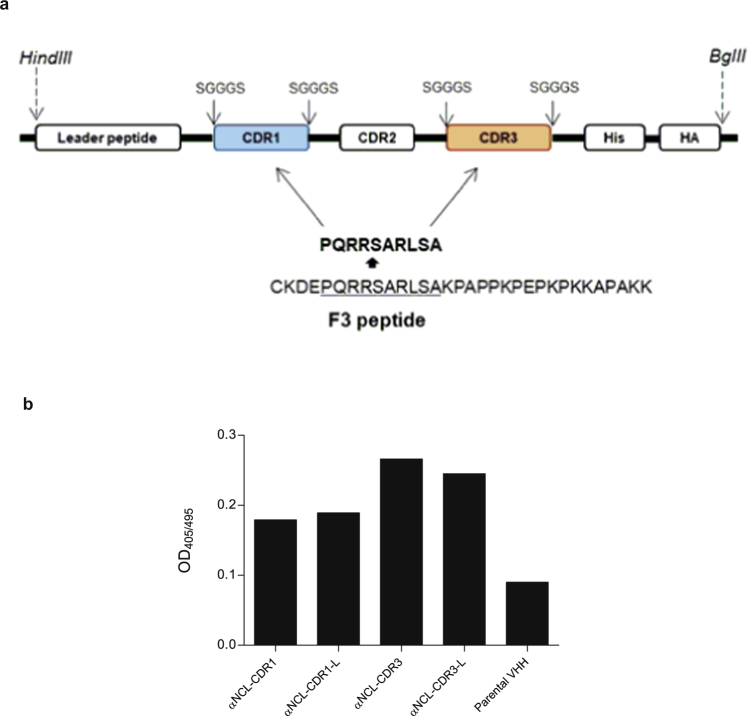
Development of different VHH constructs and binding to human nucleolin. (**a**) VHHs were developed by grafting a nucleolin-binding peptide sequence, either onto CDR1 or CDR3, with or without flanking linkers, at the end of the grafted CDR. (**b**) Binding of the generated VHHs, and the corresponding parental VHH, to human nucleolin, following 1 h incubation at 37 °C. Results are from a representative experiment.

Novel VHHs were developed upon grafting a 10-amino acid sequence, derived from the nucleolin-binding F3 peptide, onto either CDR1 or CDR3 of a parental VHH (anti-human TNF-α^25^), giving rise to two different VHHs (αNCL-CDR1 VHH and αNCL-CDR3 VHH). In addition, a variant of this sequence, flanked by the linker SGGGS at both ends, was also grafted onto each CDR, originating αNCL-CDR1-L VHH and αNCL-CDR3-L VHH (Fig. 1a). The incorporation of these flanking linkers aimed at conferring higher conformational flexibility to the CDR loop, thus improving antigen binding and recognition^26,27^. All the new generated anti-nucleolin VHH fragments bound to nucleolin, regardless of the CDR grafted, with the αNCL-CDR3 VHH and αNCL-CDR3-L VHH presenting the highest extent. The nucleolin-binding VHHs grafted onto CDR1 or CDR3 presented a 2- or 3-fold increased binding to nucleolin, respectively, relative to the parental VHH, without engraftment of the F3 peptide-derived sequence (Fig. 1b). This supported the involvement of the F3 peptide-derived sequence on the observed binding of the anti-nucleolin VHH constructs.

### Binding of anti-nucleolin VHHs to cancer cells

**Figure 2:**
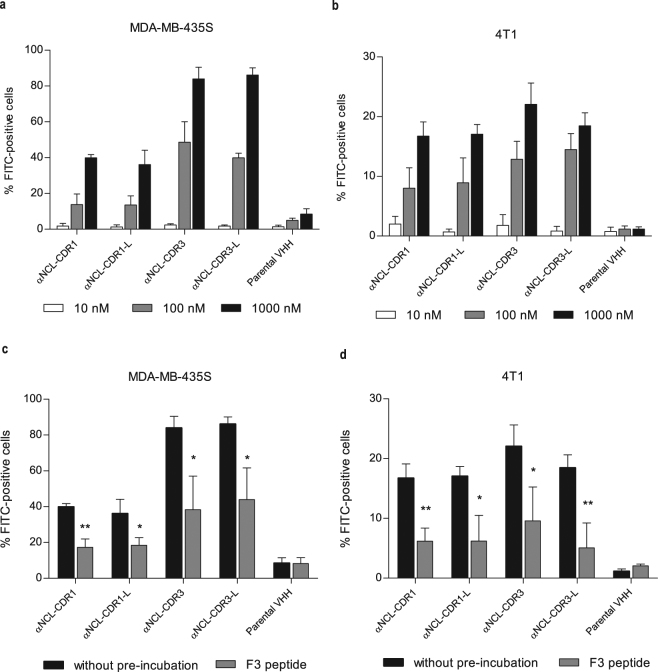
Binding of different VHH constructs to nucleolin-overexpressing cells. One hundred thousand of MDA-MB-435S (**a**,**c**) or 4T1 (**b**,**c**) cells were incubated with parental VHH or VHHs grafted with a nucleolin-binding peptide sequence either onto CDR1 or CDR3, with (αNCL-CDR1-L or αNCL-CDR3-L, respectively) or without (αNCL-CDR1 or αNCL-CDR3, respectively) flanking linkers at the end of the grafted CDR, for 45 min at 4 °C. Binding was assessed with a final incubation with anti-HA-FITC antibody and further analysis by flow cytometry. Competitive inhibition assays were also performed for each cell line (**c**,**d**), upon pre-incubation of the cells with 75 µM F3 peptide (gray bars), for 30 min at 4 °C. A control without competitive inhibition was performed (black bars). Data represent the mean ± SD of three independent experiments, performed in duplicate. Differences in binding among the VHHs tested were evaluated by one-way ANOVA followed by Tukey’s test. Differences in binding, with or without pre-incubation with F3 peptide, were evaluated by Student’s t-test (*p < 0.05, **p < 0.01, ***p < 0.001).

The binding capacity of anti-nucleolin VHHs was assessed with human and mouse cancer cells (MDA-MB-435S and 4T1, respectively)^28,29^. A concentration-dependent binding of all constructs was observed (Fig. 2a,b). In the case of MDA-MB-435S cells, a significant difference on the binding, at 100 nM, of αNCL-CDR3 (p < 0.01) or αNCL-CDR3-L (p < 0.001) relative to αNCL-CDR1 and αNCL-CDR1-L or to the parental VHH (p < 0.001), was observed. This trend, favouring a higher binding extent of the construct grafted onto CDR3, relative to CDR1, was further confirmed at 1000 nM of incubated protein (p < 0.001). At this concentration, a significant difference between the binding of α-NCL-CDR1 VHH or α-NCL-CDR1-L VHH and the parental VHH (p < 0.001) was also observed. At the highest concentration tested, CDR1- and CDR3-grafted VHHs bound to a percentage of cells of approximately 40% and 80%, respectively, whereas the parental VHH bound to less than 10% of the cells (Fig. 2a). The profile of binding of anti-nucleolin VHH constructs to 4T1 cancer cell line, relative to the parental VHH (p < 0.01 for CDR3-grafted VHHs, at 100 nM and p < 0.001 for all anti-nucleolin VHHs, at 1000 nM), was similar to the one reported for MDA-MB-435S (Fig. 2b). The difference in terms of binding arising from peptide grafting either onto CDR1 or CDR3, was not so evident as for MDA-MB-435S, and a decrease on the overall extent of binding of anti-nucleolin VHH was observed, of up to 4-fold, depending on the protein concentration.

To confirm that nucleolin was involved in the observed binding of the generated VHHs to these cancer cells, a competition assay was performed, by pre-incubating the cells with the F3 peptide. This pre-incubation reduced the extent of binding of the anti-nucleolin VHHs by at least 50% in both cell lines (Fig. 2c,d), suggesting that nucleolin was involved in the binding of the novel VHHs.

### Cytotoxicity of anti-nucleolin VHHs against cancer cells

**Figure 3:**
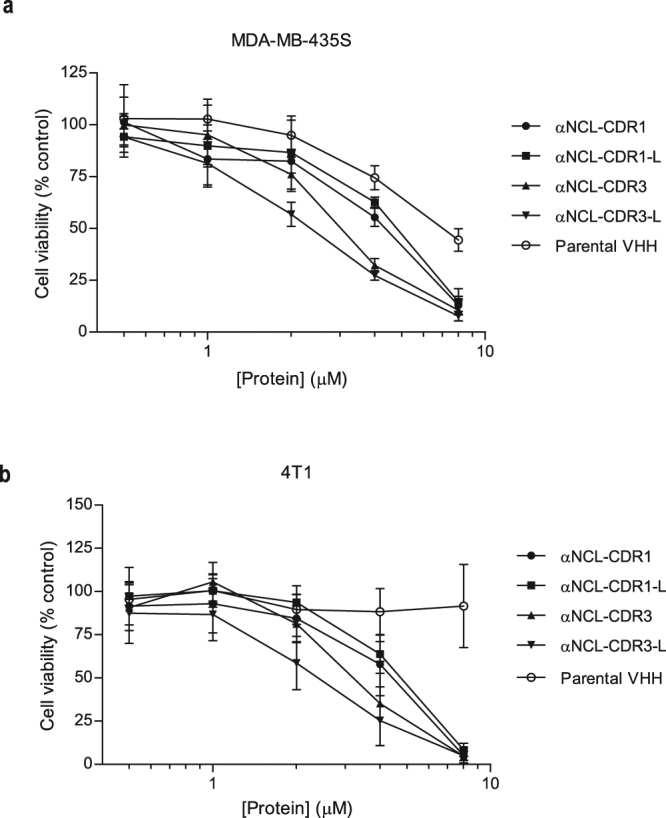
Cytotoxicity of anti-nucleolin VHHs against nucleolin-overexpressing cancer cells. Different VHH constructs, grafted with a nucleolin-binding peptide sequence either onto CDR1 or CDR3, with (αNCL-CDR1-L or αNCL-CDR3-L, respectively) or without (αNCL-CDR1 or αNCL-CDR3, respectively) flanking linkers at the end of the grafted CDR, were incubated with (**a**) MDA-MB-435S or (**b**) 4T1 cancer cells, at concentrations up to 8 µM, for 72 h at 37 °C. The parental VHH, without the targeting component to nucleolin, was included as control. In the end of the incubation, cytotoxicity was assessed by the MTT assay. Data represent the mean ± SD of at least three independent experiments, performed in duplicate. Differences in cytotoxicity among the tested VHHs were evaluated by one-way ANOVA followed by Tukey’s test (*p < 0.05, **p < 0.01, ***p < 0.001).

All anti-nucleolin VHHs presented concentration-dependent cytotoxicity against MDA-MB-435S and 4T1 cells, in the micromolar range (Fig. 3).

In the case of MDA-MB-435S cells, differences relative to the parental VHH became evident at 4 µM, with cell viability reduced to 60% (p < 0.001 for αNCL-CDR1; p < 0.01 for αNCL-CDR1-L) or 30% (p < 0.001) by the proteins grafted onto CDR1 or CDR3, respectively (corresponding to 1.5- or 2.5-fold decrease of cell viability, respectively). CDR3-grafted proteins presented a 2-fold decreased cell viability compared to CDR1-grafted proteins (p < 0.001). At 8 µM, differences of activity among anti-nucleolin VHH fragments were dissipated, resulting in less than 20% of viable cells and reaching a 1.5-fold decrease of cell viability relative to the parental VHH (p < 0.001) (Fig. 3a).

The extent of decrease of cell viability achieved with CDR3-grafted VHHs against 4T1 cancer cells, was similar to the one observed against the MDA-MB-435S cells (at 4 µM, p < 0.05 and p < 0.01 for αNCL-CDR3 and αNCL-CDR3-L, respectively, and at 8 µM, p < 0.001 for all anti-nucleolin VHHs, relative to the parental VHH) (Fig. 3b). Results suggested, once again, that grafting onto CDR3 improved the cytotoxicity efficacy of anti-nucleolin VHHs (relative to grafting onto CDR1).

### Cytotoxicity of an anti-nucleolin VHH-Fc antibody against cancer cells

**Figure 4:**
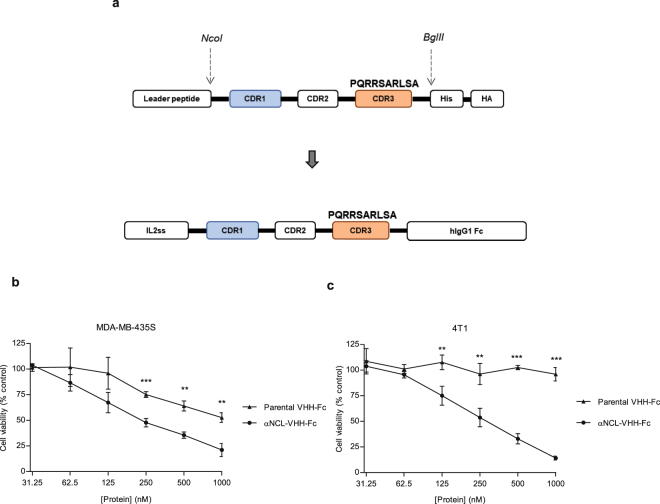
Cytotoxicity of an anti-nucleolin VHH-Fc antibody against nucleolin-overexpressing cancer cells. (**a**) An anti-nucleolin VHH-Fc antibody (αNCL-VHH-Fc) was generated by fusing αNCL-CDR3 to a human IgG1 Fc region and then incubated with (**b**) MDA-MB-435S or (**c**) 4T1 nucleolin-overexpressing cancer cells, at concentrations up to 1000 nM, for 72 h at 37 °C. The parental VHH-Fc antibody, without the targeting component to nucleolin, was included as control. In the end of the incubation, cytotoxicity was assessed by the MTT assay. Data represent the mean ± SD of three independent experiments, performed in duplicate. Differences in cytotoxicity between the VHH-Fc antibodies were evaluated by Student’s t-test (**p < 0.01, ***p < 0.001).

The αNCL-CDR3 VHH and the parental VHH, without the nucleolin-binding component, were further fused to a human IgG1 Fc region (Fig. 4a). The higher extent of cytotoxicity enabled by the αNCL-CDR3 VHH relative to the CDR1-grafted counterpart, and regardless of the presence of the flanking linkers at each end of the grafted CDR, supported the choice of the former to generate the novel αNCL VHH-Fc antibody. Improved cytotoxicity in the nanomolar range was observed with the anti-nucleolin αNCL-VHH-Fc antibody, relative to the VHH counterpart (Fig. 4b,c). A more pronounced decrease of cell viability was observed with αNCL-VHH-Fc, relative to the control antibody (parental VHH-Fc). At the highest concentration tested, αNCL-VHH-Fc led to a 1.7-fold decrease of viability of MDA-MB-435S cells relative to parental VHH-Fc.

### Antibody-dependent cell-mediated cytotoxicity (ADCC) of anti-nucleolin VHH-Fc antibody against MDA-MB-435S cells

**Figure 5:**
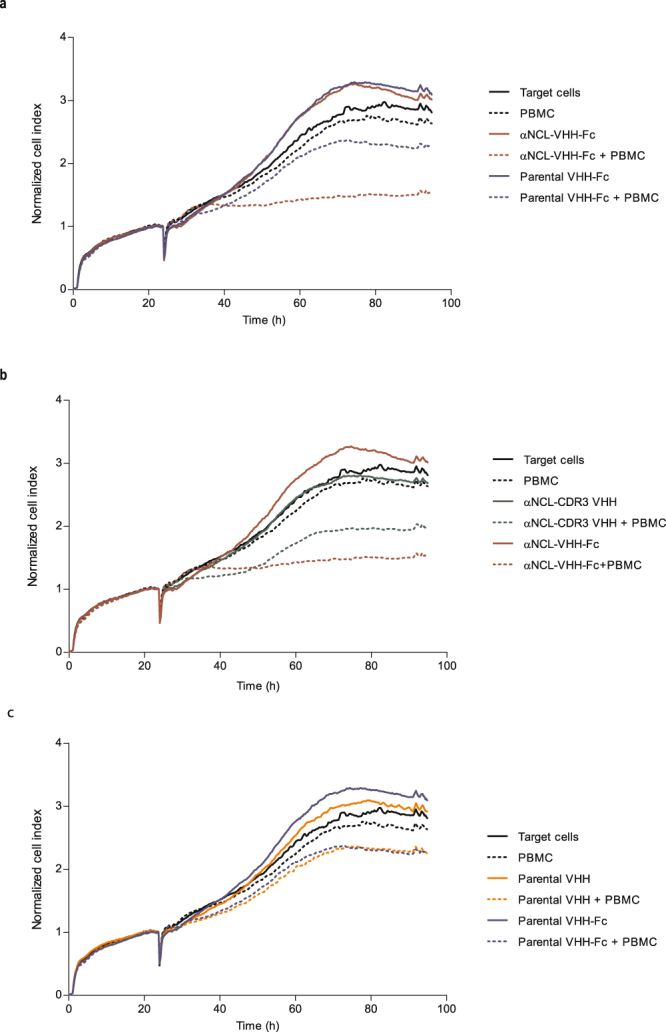
Cytotoxicity of anti-nucleolin VHHs in the presence of PBCMs, against MDA-MB-435S cells. MDA-MB-435S cells, previously cultured in a RTCA plate for 24 h, were incubated with protein, without (solid lines) or with human PBMCs (at a target cells/effector cells ratio of 1:10 or 1:5, dotted lines), for 72 h at 37 °C. Cell index, normalized to the beginning of the incubation, was measured every 15 min, using the xCELLigence system. ADCC effect of the anti-nucleolin VHH-Fc antibody (red) was further supported upon assessing its activity *versus* (**a**) 25 nM parental VHH-Fc (blue), (**b**) 50 nM αNCL-CDR3 VHH (green), (**c**) 25 nM parental VHH-Fc (blue) *versus* 50 nM parental VHH (orange). Data are from a representative experiment, performed in duplicate.

As human IgG1 Fc region enables ADCC responses^30^, we evaluated cancer cell death upon incubation with anti-nucleolin VHH-Fc antibody and effector cells (PBMCs). In the presence of PBMCs, αNCL-VHH-Fc at 25 nM enabled higher MDA-MB-435S cancer cell death than equimolar concentration of parental VHH-Fc (p < 0.001, Fig. 5a). Important to point out that this difference was completely dissipated in the absence of PBMCs (Fig. 5a). Moreover, the effect was partly dependent on the presence of the Fc region, as in the absence of the latter, the αNCL-CDR3 VHH protein enabled a lower level of cancer cell death in the presence of PBMCs (p < 0.01, Fig. 5b). The absence of ADCC activity of the parental VHH-Fc construct was further demonstrated, as it enabled similar levels of cell viability as its VHH counterpart, upon incubation with PBMCs (Fig. 5c). These results supported an overall nucleolin-dependent ADCC effect by the anti-nucleolin VHH-Fc antibody. Increased cytotoxicity of αNCL-CDR3 VHH in the presence of PBMCs, relative to αNCL-CDR3 VHH alone or parental VHH incubated with PBMCs (p < 0.01), was observed.

**Figure 6:**
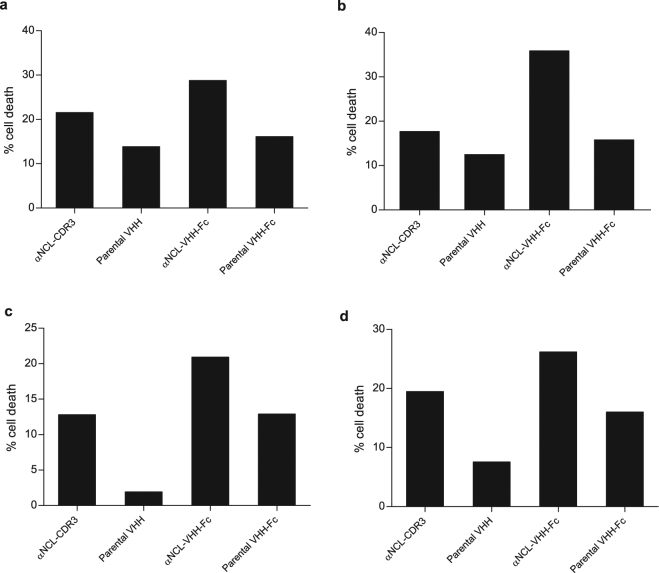
Effect of the PBMCs donor variability on the cytotoxicity of nucleolin-binding proteins against MDA-MB-435S cells. Figures a–d represent the cytotoxicity assays, performed in duplicate, with PBCMs harvested from four donors, using the xCELLigence system. MDA-MB-435S, previously cultured in a RTCA plate for 24 h, were incubated with PBMCs (at a target cells/effector cells ratio of 1:10 or 1:5) and 25 nM anti-nucleolin VHH-Fc antibody (αNCL-VHH-Fc) or the parental VHH-Fc antibody, without the nucleolin-binding component, for 72 h at 37 °C. The VHH counterparts of these antibodies (50 nM αNCL-CDR3 VHH or parental VHH) were also included as controls. Cancer cell death was calculated from the area under the curve (AUC), as described in the Methods. Differences in cytotoxicity among the tested proteins, upon incubation with PBMCs, were evaluated by repeated measures ANOVA followed by Tukey test.

The extent of cell death for each anti-nucleolin ligand and control proteins as a function of individual PBMCs donors, revealed similar profiles (Fig. 6). Increase in PBCM-dependent cell death ranged from, approximately, 1.3- to 2-fold, relative to the parental VHH-Fc and a 1.3- to 1.7-fold increase relative to the VHH counterpart (p < 0.01). Therefore, and regardless of the PBMC origin, these results supported a Fc-dependent ADCC effect of the anti-nucleolin VHH-Fc against the nucleolin-overexpressing MDA-MB-435S cancer cells.

## Discussion

Several approaches have been developed to target nucleolin-overexpressing tumours, either with targeted drug delivery using nanoparticles^21^ or nucleolin-binding agents with cytotoxic activity against nucleolin-overexpressing cells^31^. However, nucleolin targeting with antibodies remains largely unexplored, namely with those enabling an ADCC effect, a mechanism of proven therapeutic relevance for commercially available antibodies, such as cetuximab, trastuzumab or rituximab^13–18^.

The importance of ADCC as a mechanism of cell death, enabling a more favorable therapeutic outcome, has been demonstrated in several studies involving polymorphisms in the IgG1 Fc receptors-coding *FcγRIIA* and *FcγRIIIA* genes. These receptors present different expression patterns depending on the type of immune cells. FcγRIIa and FcγRIIIa/CD16a are low-affinity activating receptors for IgG1 Fc and are expressed by subpopulations of NK cells, macrophages or T cells. Some polymorphisms in these genes augment the affinity of the IgG1 Fc region towards the receptor and correlate with better clinical responses, when compared with the responses in the cohort without polymorphisms. For example, patients with non-Hodgkin lymphoma, presenting a mutation at position 158 of FcγRIIIa (where phenylalanine was replaced by valine), have been associated with complete response to rituximab, subsequent to a stronger binding to the corresponding Fc region^16,32^. In a cohort of patients with metastatic colorectal cancer, progression free survival was higher in patients with FcγRIIa-131H/H and FcγRIIIa-158V/V genotypes, regardless of the *KRAS* status^15^.

In another setting, treatment efficacy with trastuzumab was increased in patients with metastatic HER2-positive breast cancer presenting V/V or H/H genotype, which correlated with higher *ex-vivo* ADCC activity of peripheral blood mononuclear cells (PBMCs)^17,33^. In this respect, there are experimental evidences suggesting a stronger ADCC component underlying trastuzumab mechanism of action than the single interference at the level of the HER2-associated intracellular signaling pathway. In fact, in patients with HER2+ metastatic breast tumors, with complete or partial remission, upon treatment with preoperative trastuzumab, tumor infiltration of lymphoid cells was identified along with *ex-vivo* ADCC activity in the presence of PMBCs. In these patients, neither HER2 down-modulation nor changes in proliferation (as evaluated by Ki-67 staining) were observed during therapy^18^. A recent study also suggested that ADCC contributes to the treatment efficacy of trastuzumab in patients with metastatic gastric cancer. This study reported that the FcγRIIa-131H/H genotype was associated with significantly increased progression-free survival in patients treated with trastuzumab combined with chemotherapy, relative to the arm with chemotherapy alone^34^. Enhanced ADCC responses were also observed upon incubation of cetuximab with a squamous cell carcinoma of the head and neck (SCCHN) cell line, along with patient-derived NK cells, being also predictive of increased progression-free survival, and further supporting the relevance of ADCC in antibody therapy^13^.

One of the most common strategies to generate an antibody or an antibody fragment against a specific target encompass: (i) immunization of the animal model used for antibody generation, followed by spleen cell harvesting and antibody recovery, and (ii) generation of a library and selection of binders by display technologies (most frequently, phage display). However, the previous demonstration of the binding properties of specific ligands, namely peptide-based, towards a target of interest, enables the grafting of a known binding sequence onto the CDRs of the antibody^35,36^, thus bypassing the previous described time-consuming steps. In the present work, the development of anti-nucleolin VHHs relied on a grafting strategy based on the nucleolin-binding 31-amino acid F3 peptide, which had previously demonstrated promising *in vivo* tumour targeting capabilities^29,37–39^. Grafting was performed with the 10-amino acid sequence responsible for the major component of nucleolin binding by the F3 peptide^40^. No other studies have reported the use of this small sequence alone for grafting onto antibody formats.

Grafting of this 10-amino acid sequence onto CDR1 or CDR3 was justified by the fact that CDR1, and particularly CDR3, are the most important domains on antigen binding, especially in VHHs. In this antibody format, the lack of the VL domain is compensated by longer CDR1 and CDR3 domains, as well as a more exposed CDR3. These characteristics favour antigen interaction with these CDRs, mainly the latter^41,42^. In fact, some antigen interactions with VHH have been reported to occur only through CDR1 and CDR3 (as for an anti-RNase A VHH^43^) or only through CDR3 (as in an anti-carbonic anhydrase VHH^44^). Accordingly, grafting either onto CDR1 or CDR3 generated VHHs with the ability to bind nucleolin, as demonstrated with purified nucleolin and cancer cells. Competitive inhibition assays with the F3 peptide further confirmed that nucleolin was involved in the VHH binding to the cancer cell lines. The binding studies, along with the cytotoxicity experiments, supported that the modification onto CDR3 enabled a higher extent of activity relative to the exact same grafting, onto CDR1. This was in agreement with the reports showing that CDR3 usually plays a more relevant role in antigen binding, than other CDRs^41,42^. The higher binding of the developed anti-nucleolin VHHs to MDA-MB-435S cells, relative to 4T1 cells, is in accordance with the higher density of surface nucleolin of the former relative to the latter (approximately 102000 *versus* 45000 molecules, respectively; unpublished results).

Binding of the anti-nucleolin VHHs to 4T1 confirmed that these entities also bound to mouse nucleolin, which is line with the 81% homology of the protein between these two species^45^. This is somehow in contrast with the ability of D3 antibody to bind to human nucleolin, but not the one from mouse origin^46^. Binding to an epitope in a less conserved region of the protein could support the difference in binding, between the two species. The fact that the developed anti-nucleolin VHHs bound to both human and mouse nucleolin, becomes relevant when considering a future characterization of these antibodies in an *in vivo* setting, as it validates the use of either immunocompromised or immunocompetent mice models, overexpressing human or mouse nucleolin, respectively.

The reported activity of the developed anti-nucleolin VHHs did not depend on loops with increased flexibility, as enabled by SGGGS linkers inserted at the end of CDRs^26,27^. This is in contrast with the activity of camelid antibodies (HCabs), characterized by the VHH binding domains, instead of the common VH-VL. The longer and more flexible CDR3 loop of Hcabs, favors the access to clefts, characteristic of enzymes’ active site (usually not accessible to conventional antibodies)^42,47,48^. The results herein presented suggested that the epitope recognized by the developed anti-nucleolin VHHs was easily accessed, not requiring a longer or more flexible loop.

The anti-nucleolin VHHs antibodies presented cytotoxicity against the cancer cell lines tested, in the micromolar range. Although anti-nucleolin VHHs presented maximal binding to both cancer cell lines at 1 µM, cytotoxic effects at this concentration were minimal. To explain this apparent discrepancy, it is important to take into account that in highly proliferative cells, nucleolin is constantly being expressed and translocated to the surface, with a half-life of approximately 1 h^49^. In the binding experiment (Fig. 2), incubation of the cancer cells with the VHHs was performed at 4 °C for 45 min, whereas the cytotoxicity experiment (Fig. 3) was carried out at 37 °C for 72 h. Differences both in temperature and time scale of the assays, support a higher extent of newly synthesized surface nucleolin in the latter and thus the minimal cytotoxicity at 1 µM of protein, concentration at which maximal binding was observed.

The dimeric format of the anti-nucleolin VHHs (VHH-Fc) presented improved cytotoxicity, in the nanomolar range. The use of antibodies in this format might enable additional pharmacodynamics features, besides the potential ADCC. In fact, VHH-Fc are dimeric proteins, thus increasing the binding avidity and, subsequently, the cytotoxic effects, relative to VHHs alone^50^. This could support the overall increased cytotoxicity enabled by the anti-nucleolin VHH-Fc antibody (in the nanomolar range), relative to its VHH counterpart (in the micromolar range). Although the increased extent of cytotoxicity of the former could be supported by the presence of two VHH monomers^50^, the fact that even twice the concentration of VHH did not enable an effect as strong as the VHH-Fc counterpart, suggested that other mechanisms are likely to contribute to this effect. In this respect, the possible simultaneous binding of the VHH-Fc to two surface nucleolin molecules could justify the referred difference on cytotoxicity. A similar observation has been reported for cetuximab and its Fab counterpart, with the former presenting higher cytotoxicity than the latter, at concentrations of 66 and 132 nM, respectively. However, a Fab2′ fragment (bivalent) counterpart presented similar cytotoxicity to cetuximab, suggesting that the higher cytotoxicity enabled by the IgG and Fab2′ antibodies, relative to Fab, derived from their ability to simultaneously bind to two EGFR molecules^51^.

The fact that parental VHH-Fc also presented cytotoxicity against MDA-MB-435S cells (although significantly lower than the anti-nucleolin VHH-Fc) could be associated with the anti-(human) TNF-α binding of non-grafted CDRs^52,53^. In fact, some cancer cell lines (including MDA-MB-435, the parental cell line of MDA-MB-435S) have been reported to express the transmembrane form of TNF-α, tmTNF-α^54^. The absence of binding to mouse 4T1 cells was in agreement with the lack of cross-reactivity between parental VHH and TNF-α mouse counterpart^25^.

The anti-nucleolin VHH-Fc antibody presented increased effects relative to other agents targeting nucleolin, including the AS1411 aptamer and the HB-19 and N6L pseudopeptides, which impacted cancer cell viability in the micromolar range^6,10,11^. Although the extent of this effect was lower than the one observed with the anti-nucleolin scFv 4LB5 (IC_50_ values in the low nanomolar range, 3–58 nM^23^), the anti-nucleolin antibody herein presented, demonstrated ADCC capacity.

Several molecules, including EGFR-targeting agents, trigger immune responses even in the absence of a Fc domain (and thus, in an ADCC-independent mechanism)^55,56^. For this reason, in the ADCC assays, a control using the nanobody counterpart (without Fc domain) was included. The increased MDA-MB-435S cell death associated with the presence of PBMCs enabled by an anti-nucleolin VHH (grafted onto CDR3), relative to the parental VHH, suggested a Fc-independent immune response as an additional component of the mechanism of action of this protein. However, this effect was decreased relative to incubation the VHH-Fc counterpart (in the presence of PBMCs), confirming the ADCC capacity of the anti-nucleolin VHH-Fc.

Importantly, other nucleolin-targeting agents, such as HB-19 and N6L, have been reported to bind not only to nucleolin, but also to nucleophosmin and sulphated glycosaminoglycans^8,57^. In fact, nucleolin exists in a complex with nucleophosmin^58^ and interacts with sulphated glycosaminoglycans^59^, as well as with proteins, including ErbB receptors and Ras^60,61^. These reports highlighted the complexity of nucleolin interactions and thus of the mechanistic complexity associated with targeting strategies towards this protein. As such, the involvement of other proteins, besides nucleolin, in the activity of the anti-nucleolin VHH/VHH-Fc herein described, should not be ruled out.

In summary, herein it has been reported for the first time, and to the authors best knowledge, the development of nanobodies targeting nucleolin, which further fused to a human IgG1 Fc fragment, enabled ADCC activity. The latter (αNCL-VHH-Fc) could in fact emerge as a novel class of therapy against nucleolin-overexpressing tumours.

## Methods

### Reagents

Human recombinant nucleolin was from Abnova (Taiwan) and F3 peptide (KDEPQRRSARLSAKPAPPKPEPKPKKAPAKK) was custom synthesized by Genecust (Luxemburg). All other chemicals were of analytical grade purity.

### Cell culture

MDA-MB-435S (ATCC^®^ HTB-129^TM^, USA) and 4T1 (ATCC^®^ CRL-2539^TM^, USA) cell lines were cultured in RPMI-1640 (Lonza, Switzerland) and HEK293T cells (ATCC^®^ CRL-3216^TM^, USA) were cultured in Dulbecco’s Modified Eagle’s Medium (DMEM, Lonza, Switzerland). Both media were supplemented with 10% (v/v) heat-inactivated FBS (HyClone, USA), 2 mM of L-glutamine (Lonza, Switzerland) and 1% (v/v) Pen/Strep/Fungiezone solution (HyClone, USA). Cells were maintained at 37 °C in a humidified atmosphere of 5% CO_2_.

### Production of nucleolin-binding VHH

To generate a nucleolin-binding VHH, CDR1 or CDR3 domains of a parental VHH (anti-human TNF-α^25^) were grafted with the nucleolin binding F3 peptide-derived 10-aminoacid sequence PQRRSARLSA, giving rise to two different nanobodies (αNCL-CDR1 VHH and αNCL-CDR3 VHH). In addition, a variant of this sequence, flanked by the linker SGGGS at both ends, was also grafted onto each CDR, originating the VHHs αNCL-CDR1-L VHH and αNCL-CDR3-L VHH. Grafting was performed by PCR and, upon digestion of the VHH fragments with the enzymes HindIII and BglII (Thermo Scientific, USA), these were inserted onto a pT7 vector with both HA and His tags. For protein expression, performed in *E. coli* BL21 (DE3) cells, an overnight-grown culture containing the VHH of interest was diluted to 1:100 in SB medium with ampicillin (100 mg/L) and grown at 37 °C, 220 rpm, until reaching an OD between 0.7 and 0.9, at 600 nm. Protein expression was induced with 1 mM of isopropyl β-D-1-thiogalactopyranoside (IPTG, ThermoScientific, USA), for 16 h, 140 rpm. The culture was then centrifuged, resuspended in binding buffer (50 mM phosphate buffer, 300 mM NaCl, 40 mM imidazole, pH 7.0) with EDTA-free protease inhibitor cocktail (Roche, Switzerland) and sonicated. Protein was purified from the soluble fraction using HiTrap Chelating HP columns (GE Healthcare, UK).

### Production of nucleolin-binding VHH fused to Fc domain

An anti-nucleolin VHH-Fc antibody (αNCL-VHH Fc) was obtained by cloning the VHH sequence of αNCL-CDR3 VHH in the pFuse-hIgG1-Fc2 vector, upon digestion with NcoI and BglII (ThermoScientific, USA). A control antibody (parental VHH-Fc) was also generated, by cloning the parental VHH in the same vector. These were then transfected into HEK293T cells using the calcium phosphate transfection method^62^. Protein purification was performed using Pierce Chromatography Cartridges Protein A columns (Thermo Scientific, USA) and desalting was performed using disposable PD-10 columns (GE Healthcare, UK). The protein was then concentrated (Vivaspin 500, 50 kDa cutoff, GE Healhcare, UK) and stored in 20 mM HEPES, 100 mM NaCl, NaCl, 5% (v/v) glycerol, pH 8.0.

### Binding assessment of nucleolin-binding VHHs towards nucleolin protein by enzyme-linked immunosorbent assay (ELISA)

Ninety-six-well plates (Corning Costar, USA) were coated with 100 ng (19.4 nM) of human nucleolin in 50 mM sodium carbonate buffer, pH 9.6, at 4 °C, overnight, and nonspecific binding sites were blocked with 3% (w/v) BSA in PBS for 1 h, 37 °C. Each nanobody (120 pmol) diluted in 1% (w/v) BSA in PBS) was incubated for 1 h, at 37 °C, and detection was performed with anti-HA-peroxidase antibody (clone 3F10, Roche, diluted 1:1000 in 1% w/v BSA in PBS), using ABTS substrate (Merck Millipore, USA). Absorbance (405 nm/495 nm) was measured on Model 680 microplate reader (Bio-Rad, USA).

### Binding assessment of nucleolin-binding VHHs towards cancer cells by flow cytometry

MDA-MB-435S and 4T1 cancer cell lines, with different levels of surface nucleolin expression (approximately 102000 *versus* 45000 molecules, respectively; unpublished results), were used to evaluate binding of the nucleolin-binding VHHs. One hundred thousand cells, previously treated with dissociation buffer (Merck Millipore, USA), were incubated with VHH proteins, for 45 min at 4 °C, and then with anti-HA-FITC antibody (Y-11 sc-805, Santa Cruz Biotechnology, USA), at room temperature for 30 min. Competitive inhibition assays were performed by pre-incubating the cells with 75 µM F3 peptide, at 4 °C for 30 min, followed by incubation with 1000 nM VHH fragments, at 4 °C for 45 min. Sample acquisition and analysis were performed using Guava easyCyte 5HT and the InCyte software module (Merck Millipore, USA).

### Determination of the number of nucleolin molecules at the cell surface

Estimation of the density of nucleolin’s cell surface was performed by comparing cell fluorescence (measured by flow cytometry) with standard curves prepared using Quantum™ MESF Alexa488-labeled microsphere kit (Bangs Laboratories, USA) as per manufacturer instructions, upon cell incubation with DSPE-PEG_2k_-F3 micelles and anti-nucleolin-Alexa®488. This enabled to withdraw the cell Antibody Binding Capacity, i.e. the number of bound antibody molecules per cell. Accordingly, assuming a 1:1 nucleolin:antibody binding ratio, the Antibody Binding Capacity defined the cell surface nucleolin density per cell^63^.

### Cytotoxicity of nucleolin-binding VHHs against cancer cells

Five thousand MDA-MB-435S or three thousand 4T1 cells were seeded in 96-well plates. After 24 h, cell culture medium was exchanged for fresh one, and cells were incubated with serial dilutions of VHH or VHH-Fc proteins, for a total of 72 h. Cell viability was then determined with 3-(4,5-Dimethylthiazol-2-yl)-2,5-Diphenyltetrazolium Bromide (MTT) assay^64^. Absorbance at 595 nm was measured in a microplate reader (Model 680, Bio-Rad, USA) and percentage of cell viability was calculated based on equation (1):

1 $$ \% \,{\rm{cell}}\,{\rm{viability}}=\frac{{\rm{Absorbance}}\,({\rm{untreated}}\,{\rm{cells}})-{\rm{Absorbance}}\,({\rm{treated}}\,{\rm{cells}})}{\mathrm{Absorbance}\,({\rm{untreated}}\,{\rm{cells}})}\times 100$$

### PBMC isolation and culture

Human PBMCs were isolated from buffy coats of healthy volunteers (obtained at Portuguese Institute of Blood and Transplant) by a Ficoll-Paque PLUS density gradient (GE Healthcare, UK). PBMCs were then resuspended in RPMI-1640 medium (Lonza, Switzerland), supplemented with 10% (v/v) heat-inactivated FBS (HyClone, USA), 2 mM of L-glutamine (Lonza, Switzerland), 1% (v/v) Pen/Strep/Fungiezone solution (HyClone, USA) and placed at 37 °C in a humidified atmosphere of 5% CO_2_ overnight. After this recovering step, PBMCs were counted and frozen in freezing medium (10% DMSO, v/v, in FBS) and stored at −80 °C. When needed, PBMCs were thawed and cultured in the same supplemented-RPMI-1640 medium as before. PBCMs were maintained overnight at 37 °C in a humidified atmosphere of 5% CO_2_, to decrease loss of effector capacity^65^ and only then used in the ADCC assays.

### Antibody-dependent cell-mediated cytotoxicity of nucleolin-binding VHH-Fc

The ADCC potential of the anti-nucleolin VHH-Fc antibody was tested against MDA-MB-435S cancer cells (7500 cells per well), with the xCelligence Real-Time Cell Analyzer (RTCA; ACEA Biosciences, USA). Effector/target cell ratios of 5:1 or 10:1 and 25 nM of anti-nucleolin VHH-Fc were used. Additional controls comprised cancer cells incubated only with effector cells or antibody or the VHH counterparts of the VHH-Fc antibodies. As the VHH-Fc antibodies are dimeric, the VHH fragments were added in a concentration of 50 nM.

Cell index was measured every 15 min for 96 h and the resulting curves were plotted and normalized to 1.0, matching the beginning of the incubation of PBMC and the different tested proteins with the cancer cells. Data were analysed with RTCA Software Package and cancer cell death resulting from incubation with both VHH-Fc antibody and PBMCs was calculated from the area under the curve (AUC) values, approximately between 24 to 72 h, based on equation (2):

2 $$ \% \,{\rm{cell}}\,{\rm{death}}=\frac{{\rm{AUC}}({\rm{protein}}+{\rm{PBMC}})-{\rm{AUC}}({\rm{protein}})-{\rm{AUC}}({\rm{PBMC}})}{{\rm{AUC}}({\rm{untreated}}\,{\rm{cells}})}\times 100$$

### Statistical analysis

Analysis of variance (one-way ANOVA) followed by Tukey test was performed to analyse differences among the VHHs, in terms of binding and cytotoxicity. Student’s t-test was performed to analyse differences between binding without and with pre-incubation with F3 peptide and between the cytotoxicity of the nanobody-Fc antibodies. Repeated measures ANOVA followed by Tukey test was performed to analyse differences in cell death upon incubation with PBMCs and each of the proteins, in the ADCC assay. Analyses were performed with a level of significance of 5%.

### Data availability

The datasets generated during and/or analysed during the current study are not publicly available due to patent filing but are available from the corresponding author on reasonable request.
